# The Transmission and Antibiotic Resistance Variation in a Multiple Drug Resistance Clade of *Vibrio cholerae* Circulating in Multiple Countries in Asia

**DOI:** 10.1371/journal.pone.0149742

**Published:** 2016-03-01

**Authors:** Bo Pang, Pengcheng Du, Zhemin Zhou, Baowei Diao, Zhigang Cui, Haijian Zhou, Biao Kan

**Affiliations:** 1 National Institute for Communicable Disease Control and Prevention, China CDC, Beijing, 102206, People’s Republic of China; 2 State Key Laboratory of Infectious Disease Prevention and Control, Beijing, 102206, People’s Republic of China; 3 Collaborative Innovation Center for Diagnosis and Treatment of Infectious Diseases, Hangzhou, 310003, People’s Republic of China; 4 Beijing Key Laboratory of Emerging Infectious Diseases, Institute of Infectious Diseases, Beijing Ditan Hospital, Capital Medical University, Beijing, 100015, People’s Republic of China; 5 Warwick Medical School, University of Warwick, Coventry, CV4 7AL, United Kingdom; Beijing Institute of Microbiology and Epidemiology, CHINA

## Abstract

*Vibrio cholerae* has caused massive outbreaks and even trans-continental epidemics. In 2008 and 2010, at least 3 remarkable cholera outbreaks occurred in Hainan, Anhui and Jiangsu provinces of China. To address the possible transmissions and the relationships to the 7^th^ pandemic strains of those 3 outbreaks, we sequenced the whole genomes of the outbreak isolates and compared with the global isolates from the 7^th^ pandemic. The three outbreaks in this study were caused by a cluster of *V*. *cholerae* in clade 3.B which is parallel to the clade 3.C that was transmitted from Nepal to Haiti and caused an outbreak in 2010. Pan-genome analysis provided additional evolution information on the mobile element and acquired multiple antibiotic resistance genes. We suggested that clade 3.B should be monitored because the multiple antibiotic resistant characteristics of this clade and the ‘amplifier’ function of China in the global transmission of current Cholera pandemic. We also show that dedicated whole genome sequencing analysis provided more information than the previous techniques and should be applied in the disease surveillance networks.

## Introduction

Seven cholera pandemics have been acknowledged since 1817[1]. The 7^th^ pandemic erupted in Indonesia at 1961 and reached South Asia at 1963[2], where the pathogen circulated for half a century and spread over the world in multiple transmissions[3]. In 1970s the 7^th^ pandemic reached Africa and now become one of the severe health problems there; in 1990s it reached South-America and triggered large outbreaks[1]. The most recent notable event should be the 2010 Haitian cholera outbreak, which has caused more than 700,000 cases and more than 8700 deaths[4]. Nowadays, cholera remains endemic in Asia and Africa, where sanitation facilities are poor or safe water is not available[5].

China behaved as both sink and source of Cholera pandemics[6]. The 7^th^ pandemic reached China at the same year of its eruption, which was possibly brought in during the expulsion of ethnic Chinese in Indonesia between 1960 and 1961[6]. In the next 2–3 years, China might become an ‘amplifier’ of the pandemic spread to South Asia, with thousands of observed cases. Two other peaks of cholera clinic cases were recorded in China in 1980s and 1990s[7, 8], which were both sinks of transmissions from South Asia. The number of cholera cases in China decreased dramatically since 2000, and some ‘minor’ cholera outbreaks (tens to hundreds of cases reported in each) caused by O1 serogroup strains were reported[7, 8]. For example, in 2008, from late-September to mid-November, 90 cholera cases were reported in Hainan, an island at south of China. Furthermore, in the summer of 2010 (from August to September), 38 and 19 cholera cases were reported in Anhui and Jiangsu provinces, respectively. The reported numbers of cholera cases only represented a small proportion of real case numbers since most of the cases had no etiological evidence and were not found and reported. These outbreaks continued for several weeks and multiple regions were involved, which makes them quite distinguishable from the sporadic cholera cases or outbreaks caused by dinner parties, which normally contain less than ten cases.

The origins and relationships of the outbreaks described above, as well as their roles in the global circulation of the 7^th^ pandemic, and, particularly, their relationships to the remarkable Haiti outbreak at the same year, need to be addressed. Microbial genomic analysis of the re-sequencing data provides robust evidence for both evolution and transmission research of pathogenic bacteria[3, 6, 9–12], especially when the exact epidemic information were difficult to obtain. Therefore, to analyze the genomic characteristics and possible origins of the three outbreaks in 2008 and 2010, here we sequenced the genomes of 3 isolates that were isolated during the outbreaks, and compared them with our widest possible global collection of 7^th^ pandemic *V*. *cholerae* genomes, which have been reported recently[6]. We found that, from views of both core genome and HGT elements, these three outbreaks were triggered by the transmissions from South Asia, where cholera is endemic, in an existing epidemic clade 3.B, which co-exist in the same area with a genetically independent clade 3.C, which has caused Haiti outbreak. This clade has caused outbreaks and epidemics in multiple countries and is undergoing evolution with the acquirement of new characteristics by horizontal genetic transfer.

## Materials and Methods

### Strain Collection used for PFGE analysis

A total of 47 O1 toxigenic El Tor *V*. *cholerae* strains isolated between 2008–2010 in China were selected for PFGE analysis, including 20 strains from 2008 Hainan outbreak, 16 strains from 2010 Anhui outbreak, 5 strains from 2010 Jiangsu outbreak and 6 strains from other provinces in the same time period. All of these strains were isolated from patient. The O1 serogroup was confirmed by agglutination with a specific antiserum (Denka Seiken, Japan) and by PCR amplification of a genomic sequence involved in O1 lipopolysaccharide biosynthesis[13]. The *ctxAB* genes were detected with PCR using published primers[14]. Five strains, including 3 from 2008 Hainan outbreak (VC2250, VC2255, VC2272), 1 from 2010 Anhui outbreak (AHV1003) and 1 from 2010 Jiangsu outbreak (JS4) were selected for antimicrobial susceptibility test and genome Sequencing.

### Antimicrobial Susceptibility Test

Minimum Inhibition Concentration (MIC) was utilized to determine antimicrobial susceptibility of the 5 *V*. *cholerae* isolated in 2008 and 2010 in China. The antimicrobials tested were ampicillin, chloramphenicol, sulfisoxazole, tetracycline, trimethoprim/sulfamethoxazole, doxycycline, ceftriaxone, ciprofloxacin, cefepime, imipenem, cefoxitin, gentamicin, amoxicillin-clavulanic acid, streptomycin, trimethoprim and erythromycin. *Escherichia coli* ATCC 25922 was used for quality control according to Clinical and Laboratory Standards Institute (CLSI) recommendations. Routine guidelines (CLSI) and breakpoints were used for the interpretation of MIC test values[15]. The result of antibiotics test, which were not included in CLSI, were evaluated according to EUCAST.

### Whole Genome Sequencing

DNA was prepared from 1 ml overnight cultures with the Wizard Genomic DNA Purification Kit (Promega, USA) according to the manufacturer’s instructions. Whole genome sequencing was performed on 3 genomes using an Illumina HiSeq 2000 with 250-bp paired-end library (VC2250, VC2272, JS4) (BGI, China). All the data have been submitted to the European Nucleotide Archive.

### Genome Assembly and Identification of SNPs

Short reads were assembled into contigs and scaffolds using SOAPdenovo (V2.0)[16]. MuMMER (V3.0) was used to do the whole genome alignment for all of the genomes used in this study[17]. The whole genome sequence of N16961[18] was used as the reference to call SNPs in MuMMER. Any SNPs with a quality score less than 30 were excluded. SNPs in high-density clusters or in possible recombination segments were also discarded using a previously described method[19].

### Phylogenetic and Comparative Genomics Analysis

In addition to the 3 genomes sequenced in this study as described in whole genome sequencing, 260 genomes from a previous study were included[6]. A maximum likelihood tree was constructed using SNPs obtained with method described in MEGA v5[20] and a previously sequenced strain M66_2[21] was used as outgroup to root the tree. The terminal branches of this tree were colored according to the place of isolation of the strains.

To describe the detailed variations of SXT elements carried by these 5 strains, we compared these 5 SXT sequences with all SXT sequences and complete bacterial genomes in Genbank database using BLAST. The gene features and alignments between these 5 SXTs and other 2 were displayed, which are from *V*. *cholerae* MO10 (serotype O139, accession number: AY055428) and *Alteromonas macleodii* Aegaean Sea MED64 (Accession number: NC_023045). To confirm the assembling results of our 5 SXTs, short reads were mapped to the assembling sequences and the numbers of coverage were calculated. Resistance genes were predicted both by gene annotations of references and resistance genes annotation tool based on Antibiotic Resistance Genes Database (ARDB)[22]. The detailed structure of SXTs in all *V*. *cholerae* genomes included in this study were further compared both by comparison of assembled genomes using BLAST and short read mapping using SOAPdenovo[16].

## Results

### The O1 Cholera Outbreaks in 2008 and 2010 in China

From 1961, three cholera epidemics have been recognized based on the epidemiological surveillance, with a maximum of about 40,000 cases in the peak years[7, 8]. However, after 2005, reports of cholera decreased dramatically to less than 200 cases per year[7, 8]. Although sporadic cases were still recorded, there was no major (> 100 reported cases) O1 cholera outbreak reported in National Disease Surveillance System in China. During this period, two ‘medium’ level outbreaks of O1 cholera were noticed with >20 identifiable cases: in 2008, from late-September to early-November, 68 laboratory-confirmed cases (42 cases in Danzhou and 26 cases in Haikou) of cholera were reported in Hainan province. And in 2010, from mid-August to early-September, 38 laboratory-confirmed cases (37 cases in Mengcheng) of cholera were reported in Anhui province, while another 19 cases (15 cases were laboratory-confirmed while 4 cases were clinically diagnosed) were reported in early-September in a middle school in Huai-an, Jiangsu province, which only about 300 kilometers away from the earlier outbreak. No death was reported during both outbreaks. Strains isolated in Danzhou and Haikou of Hainan province in 2008 showed identical pulsed-field gel electrophoresis (PFGE) pattern (KZGN11O1.CN0724), and strains isolated in Anhui and Jiangsu provinces in 2010 also exhibited another identical PFGE pattern (KZGN11O1.CN0769), which has 2 bands differences with the pattern of the strains in 2008 (S1 Fig).

### Phylogenetic Analysis of the Chinese Outbreak Strains with the Genome Sequenced 7^th^ Pandemic Strains

**Fig 1:**
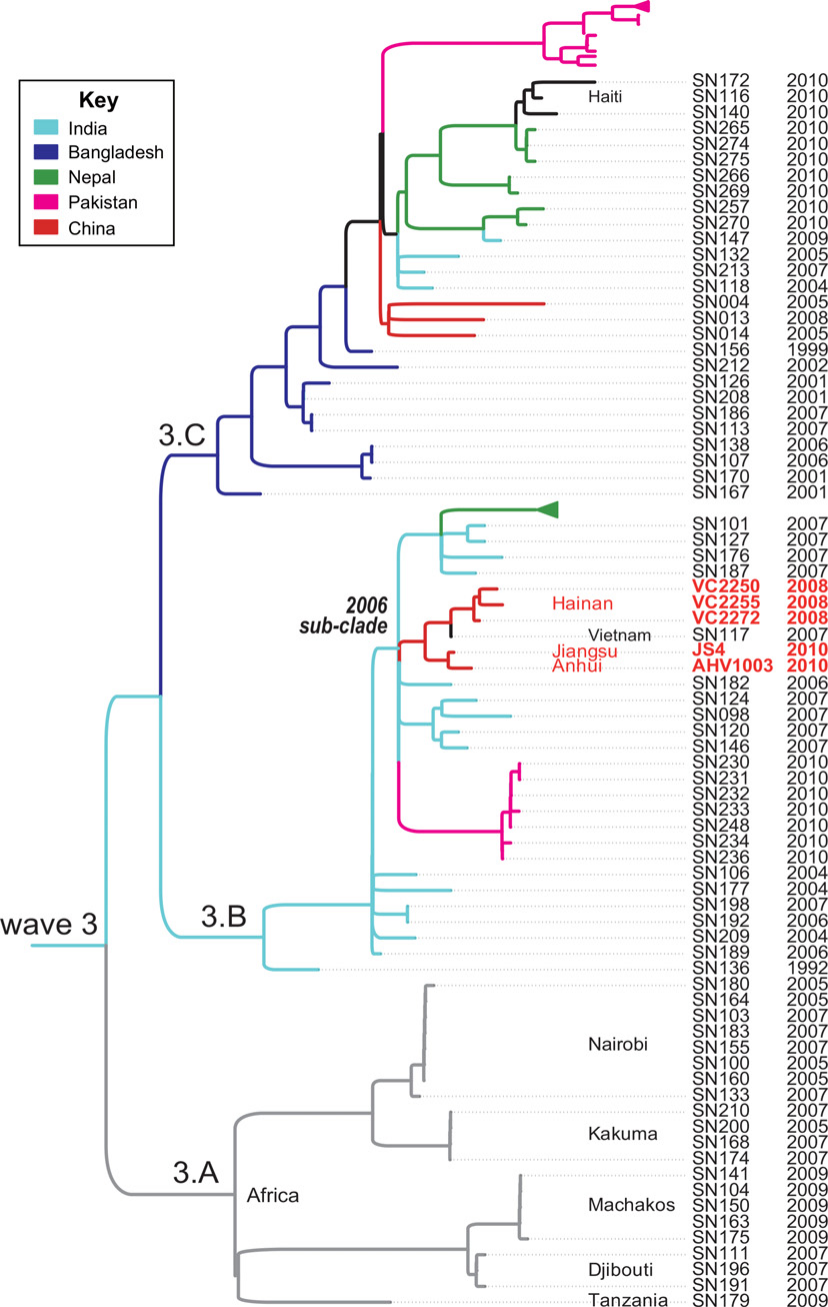
Maximum-likelihood phylogenetic tree of *V*. *cholerae* in wave 3 based on SNPs across the whole core genome, excluding possible recombination regions. The wave and clade designation is same to Mutreja et al. paper[3] and our previous study[6]. The labels of the strains (SNXXX) in wave 3 were same to our previous study[6] except for the 5 strains isolated in 2008 and 2010 China cholera outbreaks. The pre-seventh pandemic isolate M66 was used as an outgroup to root the tree. The terminal branches are colored according to the areas where the strains were isolated.

In our previous study[6], 2 strains (VC2255 from the middle of 2008 Hainan outbreak, AHV1003 from 2010 Anhui outbreak) were sequenced. In order to get information of 2010 Jiangsu outbreak and more details from 2008 Hainan outbreak, another 3 outbreak strains (Hainan 2008: VC2250 and VC2272 isolated during the early and late stage of the outbreak; Jiangsu 2010: JS4) were selected for whole genome sequencing in this study (Table A in S1 File). All of these 3 sequenced strains were assembled into less than 210 scaffolds. We compared these genomes with our widest global collection of 260 genomes of epidemic O1 strains[6] and identified 3,970 SNPs in the non-recombinant, non-mobile, non-repetitive core genome, which were then used to calculate the Maximum-Likelihood phylogenetic phylogeny (S2 Fig). The previously described waves[3] and clades[6] were also identified in the phylogeny. Clade 3.A is an Africa specific clade which represent one single transmission event from South Asia to Africa. The remaining two clades, 3.B and 3.C co-exist in 4/6 countries and circulate in the South Asia for decades. Both clades have transmitted to China in the last ten years. The causative pathogen of the Haiti 2010 outbreak was from a Nepal cluster in 3.C, which is consistent with previous reports[11, 23]. The 3 Hainan 2008 outbreak strains form a tight sub-cluster and are differed by only 5 identifiable SNPs in the core genome (Fig 1). The isolates from Anhui and Jiangsu 2010 outbreaks constitute another cluster and differentiate from the Hainan sub-cluster by only 3 SNPs.

Notably, The five Chinese isolates were clustered with a strain (SN117, namely IB4122) isolated in a Vietnam outbreak in 2007[24]. SN117 (IB4122) was differed from the Hainan cluster by only 4 SNPs. This indicates that the Vietnam 2007 outbreak and the Hainan 2008 outbreak probably share a common source, or the Vietnam might be the progenitor of the Hainan 2008 outbreak. The two areas are geographically separated only by Beibu Gulf. The ocean was a major track of transmission of *V*. *cholerae* and probably played a role in these two outbreaks.

### Variation in the SXT Element and other Mobile Elements in the Genomes of the Outbreaks

**Fig 2:**
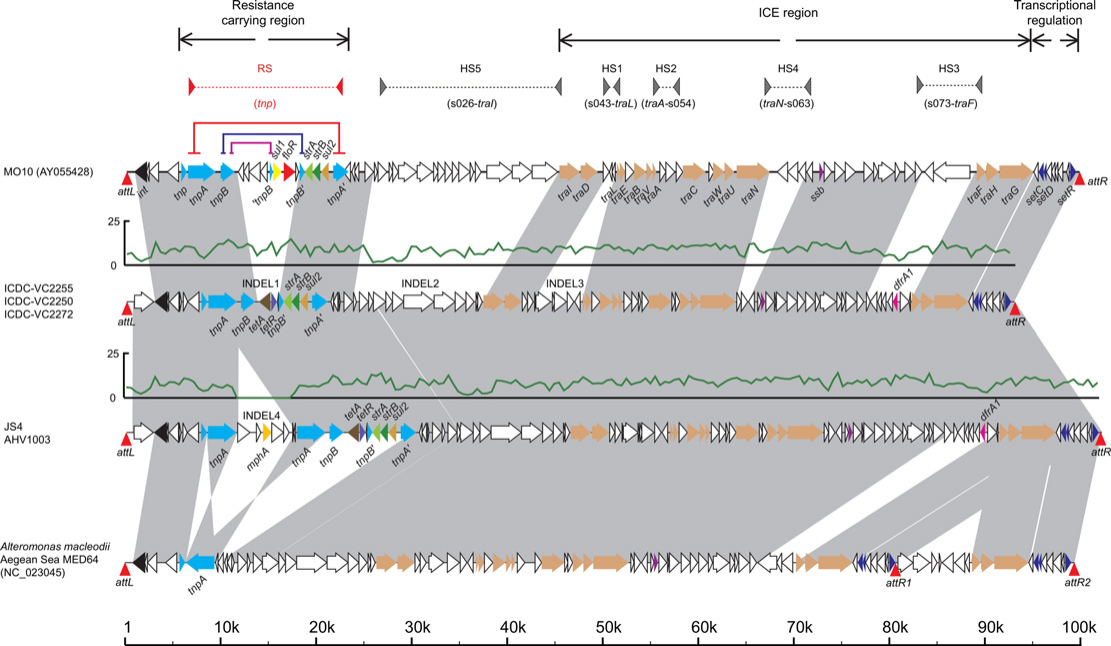
Sequence comparisons of SXT elements in O139 strain MO10, the 5 Chinese outbreak strains and *Alteromonas macleodii* Aegaean Sea MED64. Arrows with a black line represent the SXTs and CDSs, and the gray blocks represent the alignments. The colored arrows represent different types of genes (black: *int*, light blue: *tnp*, brown: *tra*, purple: *ssb*, dark blue: *set*, and others for resistance genes). The *attL* and *attR* sequences located on both ends of each SXT are displayed with red triangles. The read coverages in SXTs from the Chinese strains are displayed using green curves. Five hotspots are represented by gray triangles linked by gray dashed lines. The highly recombined region between *tnp* genes carrying resistance genes is marked using red triangles linked by red dashed lines. Three pairs of direct repeat sequences are represented by red, dark blue and purple lines. The scale at the bottom indicates the locations of the sequences.

Three specific segments spanning approximately 2kb (INDEL 1), 4kb (INDEL 3) and 13kb (INDEL 2) were uniquely identified in a sub-clade, which we arbitrarily named 2006 sub-clade (Fig 1). This sub-clade contains all five recent Chinese isolates as well as other 44 recent isolates from other countries. These segments are potential genetic markers for this sub-clade. The INDEL 1 encodes tetracycline resistance genes *tetA* and *tetR*, while INDEL 2 and 3 segments are highly similar with hypothetical genes with unknown functions in the genome of *Alteromonas macleodii* Aegean Sea MED64 (Fig 2). All three insertions are located in the sulfamethoxazole and trimethoprim (SXT) element, which was firstly discovered in *V*. *cholerae*[25].

We re-assembled the intact SXT of five Chinese outbreak strains (2008 Hainan outbreak: VC2250, VC2255, VC2272; 2010 Anhui outbreak: AHV1003; 2010 Jiangsu outbreak: JS4) by direct sequencing of PCR products that spanning gaps between contigs. When we compared their SXT regions with the SXT regions from *V*. *cholerae* O139 strain MO10 and *A*. *macleodii* MED64 (Fig 2), all STX regions from the Chinese strains were more similar to the STX region in MED64 than in MO10. INDELs 2 and 3, which accounts for 17% of the STX region, are identical to MED64, which indicates a possible inter-species horizontal transfer of SXT.

Besides the *tetA* and *tetR* genes in the INDEL 1, several other antimicrobial resistance genes, such as *strA*, *strB*, *sul2* and *dfrA1* were also found in the SXT element in the 5 Chinese strains. This finding consistent with the minimum inhibitory concentration (MIC) antimicrobial susceptibility tests in these 5 strains, which are resistant to tetracycline (strains VC2250 and VC2255 were intermediate), streptomycin, sulfisoxazole and trimethoprim (Table B in S1 File).

In addition, an additional 5kb insertion in the STX was uniquely shared among the Anhui and Jiangsu 2010 outbreak strains, as well as four Indian strains. This insertion encoded an MphA protein, which is responsible for the erythromycin resistance. This is supported by the MIC test as well. Furthermore, this insertion might be responsible for the difference between the distinguished PFGE patterns in the outbreaks.

Other major mobile elements of the *V*. *cholerae* were also surveyed. All the 5 genomes of the Chinese outbreak strains possessed the intact *Vibrio* seventh pandemic island-I (VSP-I), VSP-II, *Vibrio* pathogenicity island-I (VPI-I) and VPI-II as well, no diversity was observed in VSP-I, VSP-II and VPI-II. CTX prophage is a mobile element that showed high diversity in the regulator gene *rstR*. We amplified the *rstR* gene, the *ctxB* gene and the core-region of CTX prophage of the 5 Chinese outbreak strains. Identical *ctxB* (classical type) and *rstR* (El Tor type) gene were detected in all of the 5 tested strains, which were same to the *ctxAB* and *rstR* genes in SN117 (IB4122) and other Vietnam strains in another study [24].

## Discussion

**Fig 3:**
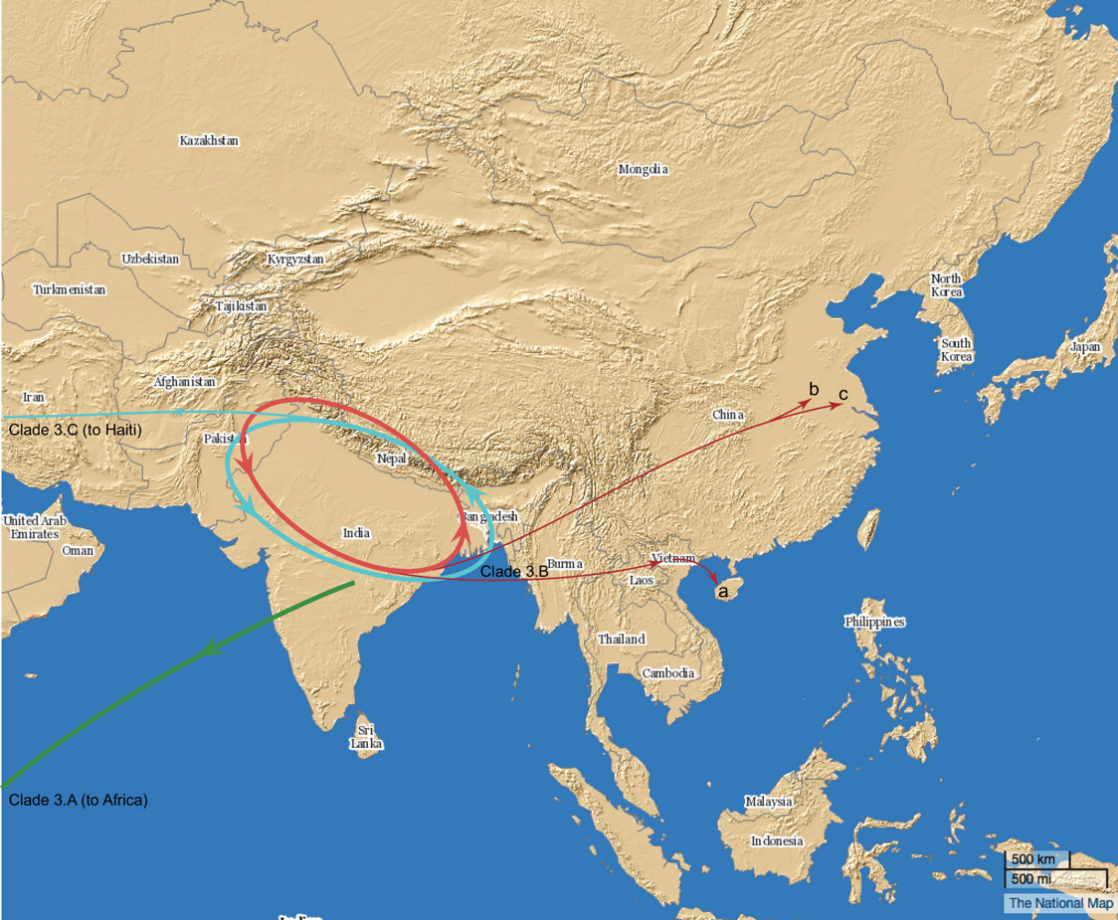
Transmission routes of the 3 *V*. *cholerae* clades in Wave 3. *V*. *cholerae* Clade 3.B and Clade 3.C circulated in South Asia. Clade 3.B reached China, (a) Hainan; (b) Anhui; (c) Jiangsu. Clade 3.C and Clade 3.A reached Haiti and Africa, respectively. The blue and red circles indicate the circulation of Clade 3.B and Clade 3.C. The map was generated with ArcGIS.

In this study, by using whole genome re-sequencing, the strains from the O1 El Tor cholera outbreaks in recent years in China were analyzed for their genomic characteristics and evolutionary relationships with the 7^th^ pandemic strains. The previous study has presented the cholera transmission in a global view[3, 6]. By combining the 3 genome sequences with the selected 260 genome sequences from previous studies[6], we found that the 5 Chinese outbreak strains lied in clade 3.B, together with a strain isolated in Vietnam in 2007. Cholera outbreaks were reported in northern Vietnam in whole 2007 (1946 cases) and early-2008 (March to April, 377 laboratory-confirmed cases) [24]. Northern Vietnam is geographically close to Danzhou and Haikou, Hainan province of China, where the Hainan outbreaks were reported. Moreover, the movements of fishermen between Hainan and Vietnam were frequent. This may contribute to the cholera transmission between these two areas, mediated most likely by human activities and/or food carriage (Fig 3). We speculate that the strains isolated in 2008 Hainan cholera outbreak and in 2007/2008 Vietnam cholera outbreak share a common ancestor. The genomic comparison and phylogenetic tree also suggested that both Anhui and Jiangsu cholera outbreaks were caused by the same strain through contaminated food or by person-to-person transmission.

Core genomic analysis has showed high performance both in evolutionary research of bacterial pathogens and the transmission of infectious disease among distant geographic regions even globally [3, 9–12, 23, 26]. However, the mobile elements and indels of genome can also be used in the meticulous discrimination of epidemic clones and source tracing. Horizontal gene transfer (HGT) is another means of genetic information transmission other than parent-to-offspring [27], which can provide insight information about the genomic variance, to describe mutations during the transmission of clones in detail. In the present study, 3 unique regions in SXT element exist in the 2006 sub-clade strains, which also correlated these strains closely and distinguished them from other cholera Wave 3 strains, hence can be used as the tracking markers to survey their spreads. Compared with the well studied and annotated SXT sequence of MO10, these indels are located at the hotspots where SXT/R391 ICEs can acquire new DNA revealed in a previous study [25]. Furthermore, although Anhui and Jiangsu 2010 outbreak strains exhibited nearly same SXT structure with Hainan outbreak strains, an extra fragment containing *mphA* gene is inserted and endow the 2010 outbreak strains with erythromycin resistance. The *mphA* gene was also detected in 4 Indian strains isolated in 2007. Besides the use in human, erythromycin is used as a veterinary drug in animal husbandry and aquaculture, therefore, this could be an example in which the element involved in survival advantage is obtained during the transmission and evolution of the strains in the same genetic clone, through the HGT from other strains carrying *mphA* gene, possibly in the medical treatment of animal husbandry or aquaculture. And, it should be inferable that adding erythromycin into the enrichment and selective culture media may increase the efficiency of the pathogen isolation from the environmental and food samples, by inhibiting the interference of the competitive bacteria, to promote the source tracing in the epidemic caused by such clone. The indels and HGT elements comparison, other than core genome data, provides more resolution to differentiate genetically close related strains. However, the explanation of mobile element data should be under the framework of similar core-genome since recombination may influence the inference of genetic relationship of the strains based on mobile element data[28]. Due to the quality limitation of some sequencing read data from a previous study which we can not assemble the whole genomes especially the SXT element well enough [3], we compared SXTs in 5 Chinese *V*. *cholerae* strains with complete genomes of other species, and interestingly these SXT sequences were highly similar with the one from *Alteromonas macleodii* Aegean Sea MED64 (Fig 2). Meanwhile, some *V*. *cholerae* O1 El Tor strains in Mutreja’s study seemed to carry the same type of SXTs by mapping the read data to SXTs from Chinese strains and alignments of gapped sequences (S3 Fig). These results indicated that this type of SXT could transfer horizontally both intra- and inter- species and has spread widely, which require more attention to discover the transmission. However, it is still hard to understand how can two strains differed greatly both in geography and taxonomy can share such identical fragment.

Based on the genomic and epidemic analysis in this study, we suggested that the 3 cholera outbreaks in 2008 and 2010 in China were caused by strains in clade 3.B in Wave 3, which maybe transmitted into China from South Asia. The strains in clade 3.B should be monitored because the frequent loss and gain of multiple antibiotic resistance genes and the ‘amplifying’ role of China in the global spread of cholera in the current pandemic. PFGE has showed good performance in outbreak detection and source tracking, however, it is incapable to resolve deep genetic relationships and have been questioned in other pathogens[29]. Whole genome sequencing, by both providing information on core genome and mobile elements, can reveal the genetic status of these pathogens in greater details and can be a potential candidate for the techniques integrated into the next generation disease surveillance systems.

## Supporting Information

S1 Fig PFGE analysis of 47 O1 toxigenic El Tor *V*. *cholerae* strains isolated between 2008–2010 in China. (PDF)

S2 Fig Maximum-likelihood phylogenetic tree of the test *V*. *cholerae* based on SNPs across the whole core genome, excluding possible recombination regions. (PDF)

S3 Fig Structure variation of SXT in O1 El Tor *V*. *cholerae*. (PDF)

S1 File Information of the 5 Chinese *V*. *cholerae* used in this study. (DOCX)
