# Supplementary material for: The Transmission and Antibiotic Resistance Variation in a Multiple Drug Resistance Clade of Vibrio cholerae Circulating in Multiple Countries in Asia
Source: PLoS One. 2016 Mar 1;11(3):e0149742. doi: 10.1371/journal.pone.0149742 (PMC4773069; doi:10.1371/journal.pone.0149742)
Supplement: S1 Fig — (PDF) [file pone.0149742.s001.pdf]

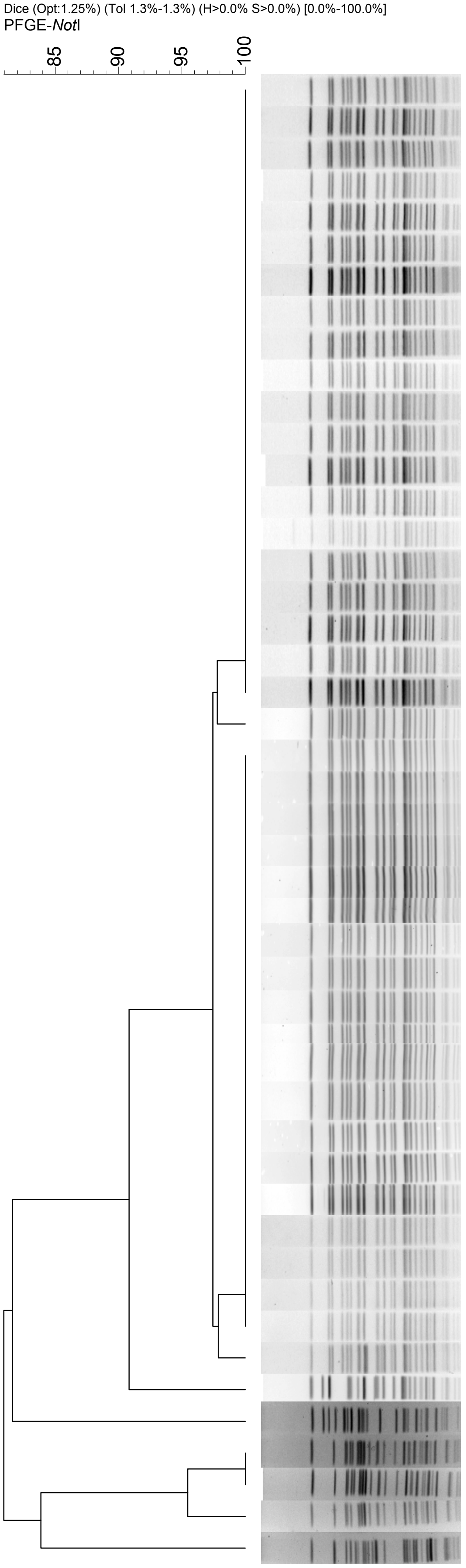

Strain ID	PFGE pattern	Isolation province	Isolation year
**ICDC-VC2250**	KZGN11O1.CN0724	Hainan	2008
ICDC-VC2272	KZGN11O1.CN0724	Hainan	2008
ICDC-VC2251	KZGN11O1.CN0724	Hainan	2008
ICDC-VC2252	KZGN11O1.CN0724	Hainan	2008
ICDC-VC2253	KZGN11O1.CN0724	Hainan	2008
ICDC-VC2254	KZGN11O1.CN0724	Hainan	2008
**ICDC-VC2255**	KZGN11O1.CN0724	Hainan	2008
ICDC-VC2256	KZGN11O1.CN0724	Hainan	2008
ICDC-VC2257	KZGN11O1.CN0724	Hainan	2008
ICDC-VC2258	KZGN11O1.CN0724	Hainan	2008
ICDC-VC2259	KZGN11O1.CN0724	Hainan	2008
ICDC-VC2260	KZGN11O1.CN0724	Hainan	2008
ICDC-VC2261	KZGN11O1.CN0724	Hainan	2008
ICDC-VC2264	KZGN11O1.CN0724	Hainan	2008
ICDC-VC2265	KZGN11O1.CN0724	Hainan	2008
ICDC-VC2266	KZGN11O1.CN0724	Hainan	2008
ICDC-VC2268	KZGN11O1.CN0724	Hainan	2008
ICDC-VC2269	KZGN11O1.CN0724	Hainan	2008
ICDC-VC2270	KZGN11O1.CN0724	Hainan	2008
**ICDC-VC2272**	KZGN11O1.CN0724	Hainan	2008
AHV1012	KZGN11O1.CN0770	Anhui	2010
AHV1001	KZGN11O1.CN0769	Anhui	2010
AHV1002	KZGN11O1.CN0769	Anhui	2010
**AHV1003**	KZGN11O1.CN0769	Anhui	2010
AHV1004	KZGN11O1.CN0769	Anhui	2010
AHV1005	KZGN11O1.CN0769	Anhui	2010
AHV1006	KZGN11O1.CN0769	Anhui	2010
AHV1007	KZGN11O1.CN0769	Anhui	2010
AHV1008	KZGN11O1.CN0769	Anhui	2010
AHV1009	KZGN11O1.CN0769	Anhui	2010
AHV1010	KZGN11O1.CN0769	Anhui	2010
AHV1011	KZGN11O1.CN0769	Anhui	2010
AHV1013	KZGN11O1.CN0769	Anhui	2010
AHV1014	KZGN11O1.CN0769	Anhui	2010
AHV1015	KZGN11O1.CN0769	Anhui	2010
AHV1019	KZGN11O1.CN0769	Anhui	2010
**JS4**	KZGN11O1.CN0769	Jiangsu	2010
JS5	KZGN11O1.CN0769	Jiangsu	2010
JS6	KZGN11O1.CN0769	Jiangsu	2010
JS7	KZGN11O1.CN0769	Jiangsu	2010
JS2	KZGN11O1.CN0772	Jiangsu	2010
HB2008001	KZGN11O1.CN1185	Hubei	2008
2010-009	KZGN11O1.CN0768	Beijing	2010
2010-010	KZGN11O1.CN0762	Beijing	2010
2010-026	KZGN11O1.CN0762	Beijing	2010
2010-006	KZGN11O1.CN0764	Beijing	2010
2010-007	KZGN11O1.CN0767	Beijing	2010
