# Supplementary figures and images for: The Transmission and Antibiotic Resistance Variation in a Multiple Drug Resistance Clade of Vibrio cholerae Circulating in Multiple Countries in Asia
Source: PLoS One. 2016 Mar 1;11(3):e0149742. doi: 10.1371/journal.pone.0149742 (PMC4773069; doi:10.1371/journal.pone.0149742)

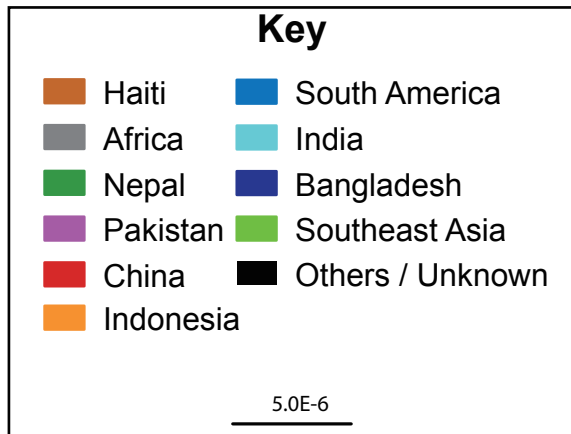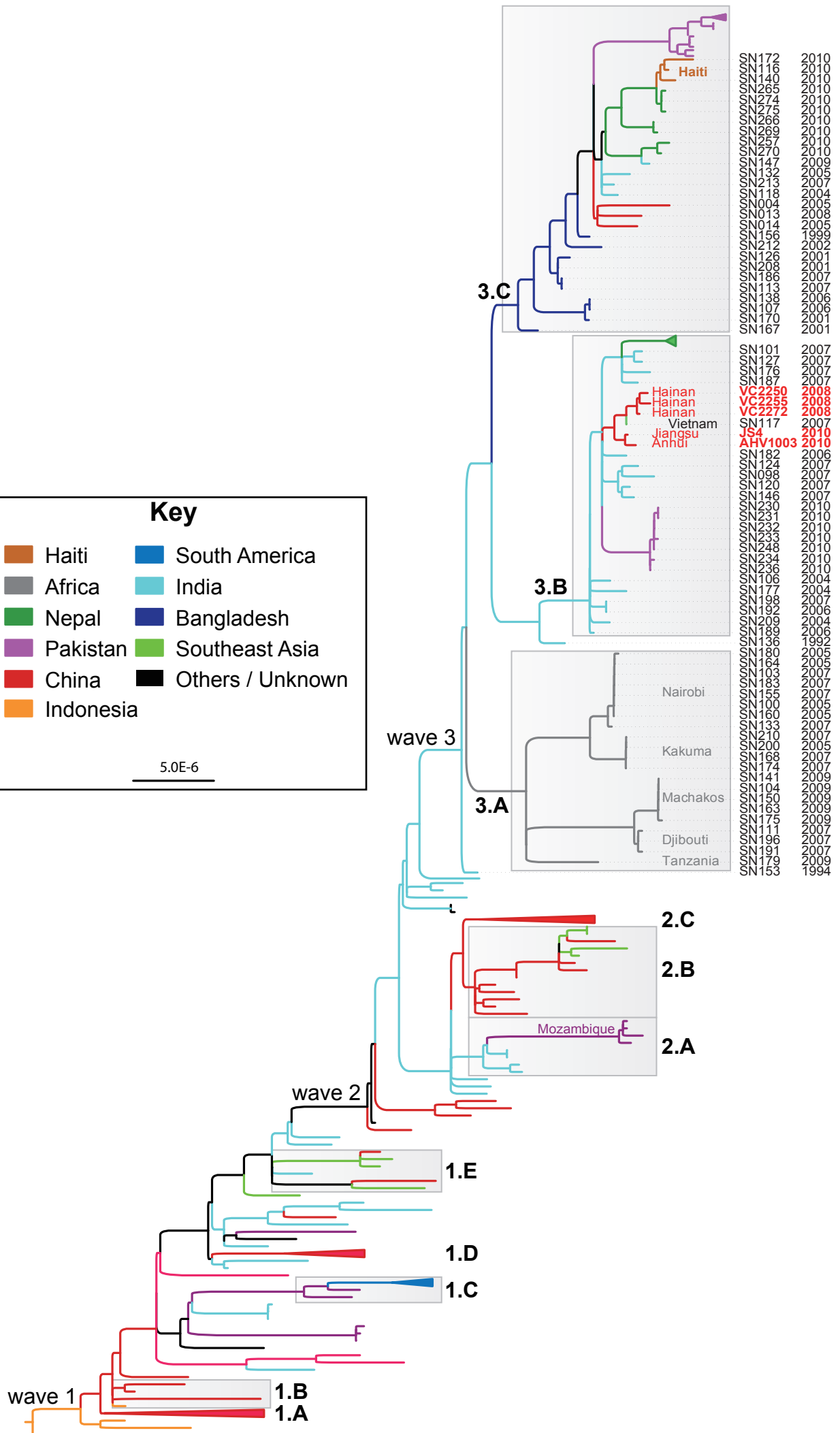

Supplement: S2 Fig — (PDF) [file pone.0149742.s002.pdf]

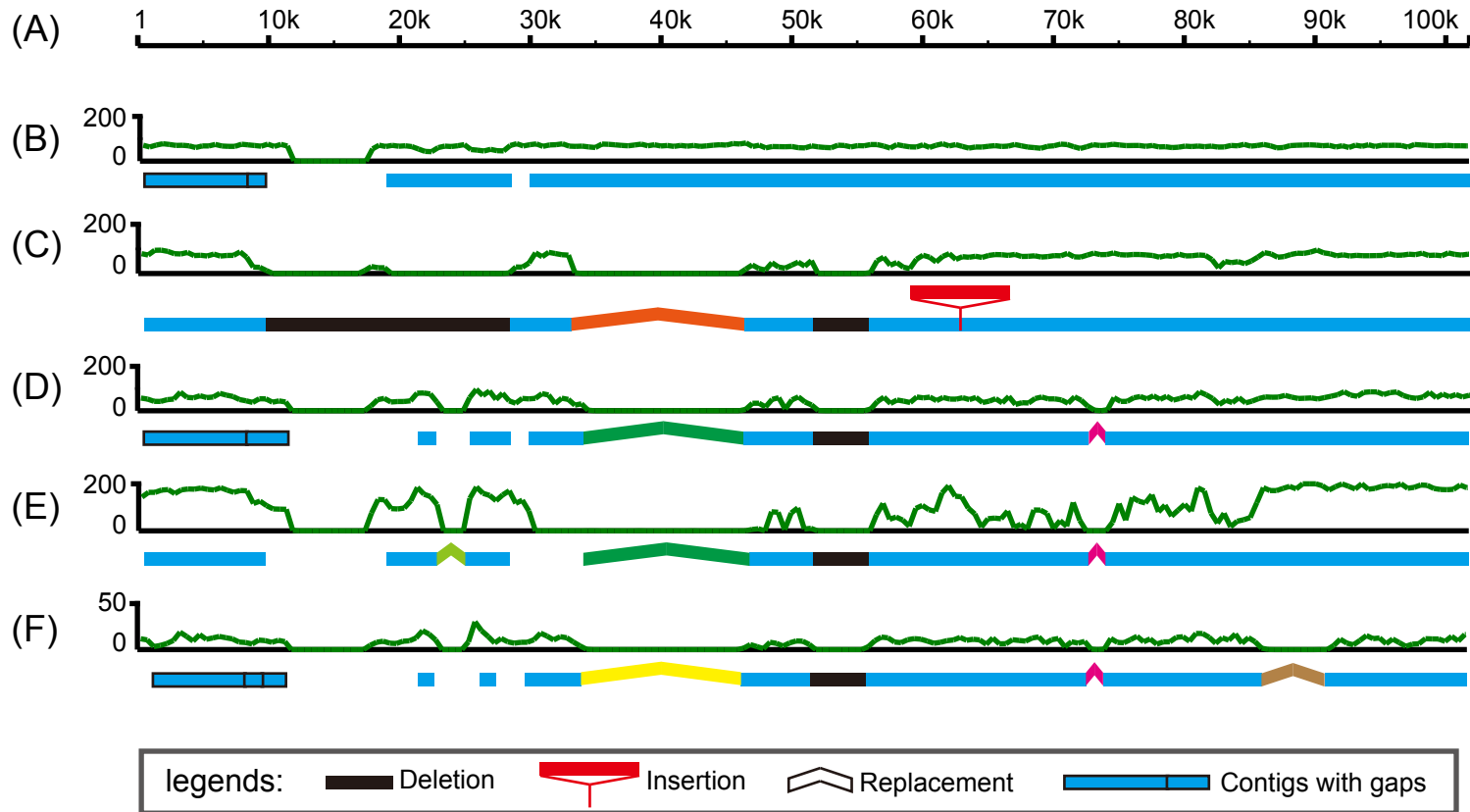

Supplement: S3 Fig — (PDF) [file pone.0149742.s003.pdf]
