# Supplementary material for: The Transmission and Antibiotic Resistance Variation in a Multiple Drug Resistance Clade of Vibrio cholerae Circulating in Multiple Countries in Asia
Source: PLoS One. 2016 Mar 1;11(3):e0149742. doi: 10.1371/journal.pone.0149742 (PMC4773069; doi:10.1371/journal.pone.0149742)
Supplement: S1 File — (DOCX) [file pone.0149742.s004.docx]

Table A Information of five Chinese *V. cholerae* in this study

| Strain | Time of Isolation | Source | PFGE Pattern |
| --- | --- | --- | --- |
| ICDC-VC2250 | 2008 | Patient | KZGN11O1.CN0724 |
| ICDC-VC2255 | 2008 | Patient | KZGN11O1.CN0724 |
| ICDC-VC2272 | 2008 | Patient | KZGN11O1.CN0724 |
| AHV-1003 | 2010 | Patient | KZGN11O1.CN0769 |
| JS4 | 2010 | Patient | KZGN11O1.CN0769 |

Table B Antibiotic Resistance profile of the 5 Chinese *V. cholerae* isolated in 2008 and 2010

| Strain | Ciprofloxacin | Erythromycin | Streptomycin | Tetracycline | Ampicillin | Sulfisoxazole | Trimethoprim sulfamethoxazole | Chloramphenicol |
| --- | --- | --- | --- | --- | --- | --- | --- | --- |
| ICDC-VC2250 | S | R | R | I | S | R | R | S |
| ICDC-VC2255 | S | R | R | I | S | R | R | S |
| ICDC-VC2272 | S | R | R | R | S | R | R | S |
| AHV1003 | S | S | R | R | S | R | R | S |
| JS4 | S | S | R | R | S | R | R | S |
